# 2-Cyano-2-methyl­propanamide

**DOI:** 10.1107/S1600536812013360

**Published:** 2012-03-31

**Authors:** Jia-Ying Xu, Wei-Hua Cheng

**Affiliations:** a College of Chemical and Biological Engineering, Yancheng Institute of Technology, Yinbing Road No. 9 Yancheng, Yancheng 224051, People’s Republic of China; b Department of Chemical Engineering, Yancheng College of Textile Technology, People’s Republic of China

## Abstract

In the crystal structure of the title compound, C_5_H_8_N_2_O, mol­ecules are linked *via* pairs of N—H⋯O hydrogen bonds, forming inversion dimers. These dimers are linked *via* pairs of N—H⋯H hydrogen bonds into zigzag chains propagating along [101].

## Related literature

For the synthesis of the title compound, see: Zhang *et al.* (2011 ▶). For standard bond-length data, see: Allen *et al.* (1987 ▶).
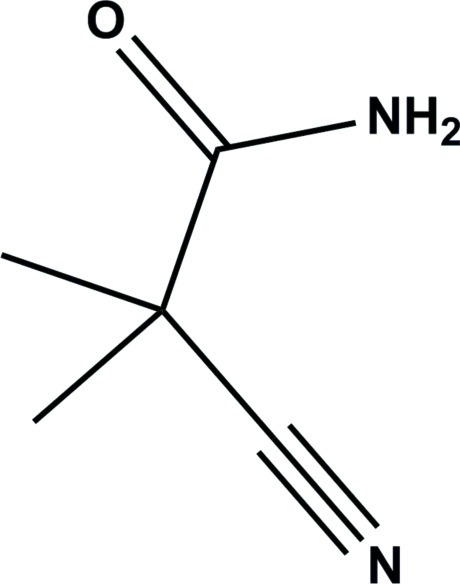


## Experimental

### 

#### Crystal data

C_5_H_8_N_2_O
*M* *_r_* = 112.13
Triclinic, 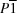

*a* = 5.8916 (12) Å
*b* = 6.4349 (14) Å
*c* = 9.1263 (19) Å
α = 95.659 (4)°
β = 102.379 (4)°
γ = 109.859 (4)°
*V* = 312.27 (11) Å^3^
*Z* = 2
Mo *K*α radiation
μ = 0.09 mm^−1^
*T* = 293 K
0.20 × 0.18 × 0.15 mm

#### Data collection

Enraf–Nonius CAD-4 diffractometer
Absorption correction: ψ scan (North *et al.*, 1968 ▶) *T*_min_ = 0.983, *T*_max_ = 0.987
1699 measured reflections
1077 independent reflections
1000 reflections with *I* > 2σ(*I*)
*R*_int_ = 0.021
3 standard reflections every 200 reflections intensity decay: 1%

#### Refinement

*R*[*F* ^2^ > 2σ(*F* ^2^)] = 0.050
*wR*(*F* ^2^) = 0.143
*S* = 1.05
1077 reflections
84 parameters
H atoms treated by a mixture of independent and constrained refinement
Δρ_max_ = 0.26 e Å^−3^
Δρ_min_ = −0.26 e Å^−3^

### 

Data collection: *CAD-4 Software* (Enraf–Nonius, 1985 ▶); cell refinement: *CAD-4 Software*; data reduction: *XCAD4* (Harms & Wocadlo, 1995 ▶); program(s) used to solve structure: *SHELXS97* (Sheldrick, 2008 ▶); program(s) used to refine structure: *SHELXL97* (Sheldrick, 2008 ▶); molecular graphics: *SHELXTL* (Sheldrick, 2008 ▶); software used to prepare material for publication: *SHELXTL*.

## Supplementary Material

Crystal structure: contains datablock(s) I, global. DOI: 10.1107/S1600536812013360/su2395sup1.cif

Structure factors: contains datablock(s) I. DOI: 10.1107/S1600536812013360/su2395Isup2.hkl

Supplementary material file. DOI: 10.1107/S1600536812013360/su2395Isup3.cml

Additional supplementary materials:  crystallographic information; 3D view; checkCIF report

## Figures and Tables

**Table 1:** Hydrogen-bond geometry (Å, °)

*D*—H⋯*A*	*D*—H	H⋯*A*	*D*⋯*A*	*D*—H⋯*A*
N1—H1*A*⋯O1^i^	0.92 (2)	2.07 (2)	2.9714 (18)	168.2 (18)
N1—H1*B*⋯N2^ii^	0.874 (18)	2.328 (18)	3.166 (2)	160.8 (19)

Symmetry codes: (i) 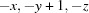; (ii) 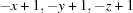.

**Fig. 1.**
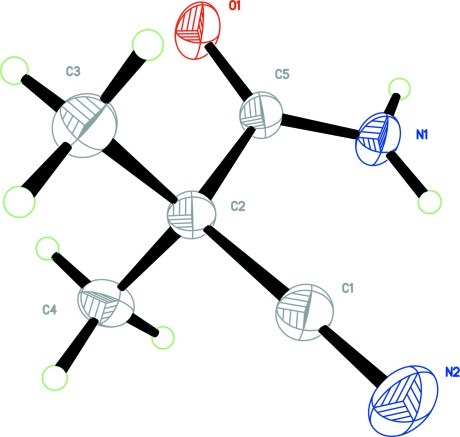
The molecular structure of the title molecule, with atom-numbering. Displacement ellipsoids are drawn at the 35% probability level.

**Fig. 2.**
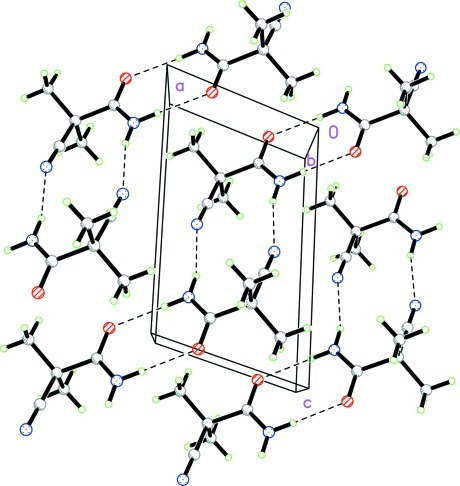
A view along the b axis of the crystal packing of the title compound. The N—H···O and N—H···N hydrogen bonds are shown as dashed lines (see Table 1 for details).

## supplementary crystallographic information

### Comment

The title compound has attracted considerable attention in drug research because of its outstanding biological activity. In recent years it has been used as an imtermediate in the synthesis of the high blood pressure rennin inhibitor, Aliskiren (Zhang *et al.*, 2011).

The molecular structure of the title compound is shown in Fig. 1. The bond lengths (Allen *et al.*, 1987) and angles are within normal ranges.

In the crystal, molecules are connected *via* pairs of N—H···O hydrogen bonds to form inversion dimers (Table 1 and Fig. 2). These dimers are connected via pairs of N—H···N hydrogen bonds resulting in the formation of zigzag chains (Table 1 and Fig. 2), propagating along direction [101].

### Experimental

The title compound was prepared by the literature procedure (Zhang *et al.*, 2011). To a solution of methyl 2-cyano-2-methylpropanoate (5 g, 39.3 mmol) in methanol (20 ml), ammonia was added slowly at room temperature. After being stirred for 18 h at the room tempreature, a yellow solid was obtained. It was dissolved in ethanol and colourless block-like crystals of the title compound, suitable for X-ray diffraction analysis, were obtained by slow evaporation of the solvent over 7 days.

### Refinement

The NH_2_ H atoms were located in a difference electron density map and refined freely. The methyl H atoms were positioned geometrically and constrained to ride on their parent atoms: C—H = 0.96 Å with *U*_iso_(H) = 1.5*U*_eq_(C).

### Crystal data

**Table:**

C_5_H_8_N_2_O	*Z* = 2
*M**_r_* = 112.13	*F*(000) = 120
Triclinic, *P*1	*D*_x_ = 1.193 Mg m^−^^3^
Hall symbol: -P 1	Mo *K*α radiation, λ = 0.71073 Å
*a* = 5.8916 (12) Å	Cell parameters from 1603 reflections
*b* = 6.4349 (14) Å	θ = 2.3–30.1°
*c* = 9.1263 (19) Å	µ = 0.09 mm^−^^1^
α = 95.659 (4)°	*T* = 293 K
β = 102.379 (4)°	Block, colourless
γ = 109.859 (4)°	0.20 × 0.18 × 0.15 mm
*V* = 312.27 (11) Å^3^	

### Data collection

**Table:**

Enraf–Nonius CAD-4 diffractometer	1000 reflections with *I* > 2σ(*I*)
Radiation source: fine-focus sealed tube	*R*_int_ = 0.021
Graphite monochromator	θ_max_ = 25.0°, θ_min_ = 2.3°
ω/2θ scans	*h* = −6→6
Absorption correction: ψ scan (North *et al.*, 1968)	*k* = −6→7
*T*_min_ = 0.983, *T*_max_ = 0.987	*l* = −10→7
1699 measured reflections	3 standard reflections every 200 reflections
1077 independent reflections	intensity decay: 1%

### Refinement

**Table:**

Refinement on *F*^2^	Secondary atom site location: difference Fourier map
Least-squares matrix: full	Hydrogen site location: inferred from neighbouring sites
*R*[*F*^2^ > 2σ(*F*^2^)] = 0.050	H atoms treated by a mixture of independent and constrained refinement
*wR*(*F*^2^) = 0.143	*w* = 1/[σ^2^(*F*_o_^2^) + (0.1061*P*)^2^ + 0.0265*P*] where *P* = (*F*_o_^2^ + 2*F*_c_^2^)/3
*S* = 1.05	(Δ/σ)_max_ < 0.001
1077 reflections	Δρ_max_ = 0.26 e Å^−^^3^
84 parameters	Δρ_min_ = −0.26 e Å^−^^3^
0 restraints	Extinction correction: *SHELXL97* (Sheldrick, 2008), Fc^*^=kFc[1+0.001xFc^2^λ^3^/sin(2θ)]^-1/4^
Primary atom site location: structure-invariant direct methods	Extinction coefficient: 2.05 (18)

### Special details

**Table:**

Geometry. All e.s.d.'s (except the e.s.d. in the dihedral angle between two l.s. planes) are estimated using the full covariance matrix. The cell e.s.d.'s are taken into account individually in the estimation of e.s.d.'s in distances, angles and torsion angles; correlations between e.s.d.'s in cell parameters are only used when they are defined by crystal symmetry. An approximate (isotropic) treatment of cell e.s.d.'s is used for estimating e.s.d.'s involving l.s. planes.
Refinement. Refinement of *F*^2^ against ALL reflections. The weighted *R*-factor *wR* and goodness of fit *S* are based on *F*^2^, conventional *R*-factors *R* are based on *F*, with *F* set to zero for negative *F*^2^. The threshold expression of *F*^2^ > σ(*F*^2^) is used only for calculating *R*-factors(gt) *etc*. and is not relevant to the choice of reflections for refinement. *R*-factors based on *F*^2^ are statistically about twice as large as those based on *F*, and *R*- factors based on ALL data will be even larger.

### Fractional atomic coordinates and isotropic or equivalent isotropic displacement parameters (Å^2^)

**Table:**

	*x*	*y*	*z*	*U*_iso_*/*U*_eq_	
O1	0.29396 (18)	0.69885 (17)	0.01288 (11)	0.0555 (4)	
N1	0.2126 (2)	0.51350 (19)	0.20281 (15)	0.0495 (5)	
N2	0.7598 (2)	0.6725 (2)	0.48798 (16)	0.0644 (5)	
C1	0.6955 (2)	0.7399 (2)	0.38232 (15)	0.0448 (5)	
C2	0.6156 (2)	0.83173 (18)	0.24687 (13)	0.0364 (4)	
C3	0.8132 (2)	0.8719 (2)	0.15614 (16)	0.0479 (5)	
H3A	0.9726	0.9721	0.2208	0.072*	
H3B	0.7658	0.9376	0.0703	0.072*	
H3C	0.8246	0.7312	0.1206	0.072*	
C4	0.5862 (3)	1.0547 (2)	0.29886 (16)	0.0494 (5)	
H4A	0.4580	1.0266	0.3521	0.074*	
H4B	0.5407	1.1176	0.2112	0.074*	
H4C	0.7418	1.1586	0.3656	0.074*	
C5	0.3573 (2)	0.66995 (19)	0.14359 (14)	0.0381 (4)	
H1A	0.059 (4)	0.429 (3)	0.138 (2)	0.069 (5)*	
H1B	0.254 (4)	0.491 (3)	0.296 (2)	0.064 (5)*	

### Atomic displacement parameters (Å^2^)

**Table:**

	*U*^11^	*U*^22^	*U*^33^	*U*^12^	*U*^13^	*U*^23^
O1	0.0468 (7)	0.0567 (7)	0.0374 (6)	−0.0025 (5)	−0.0094 (4)	0.0185 (5)
N1	0.0391 (7)	0.0511 (8)	0.0394 (7)	0.0004 (5)	−0.0051 (5)	0.0174 (5)
N2	0.0506 (8)	0.0740 (9)	0.0501 (8)	0.0094 (6)	−0.0086 (6)	0.0274 (7)
C1	0.0344 (7)	0.0472 (7)	0.0392 (8)	0.0058 (5)	−0.0033 (5)	0.0101 (6)
C2	0.0332 (7)	0.0376 (7)	0.0308 (7)	0.0087 (5)	−0.0002 (5)	0.0073 (5)
C3	0.0390 (7)	0.0570 (8)	0.0438 (8)	0.0150 (6)	0.0075 (6)	0.0102 (6)
C4	0.0487 (8)	0.0470 (8)	0.0447 (8)	0.0170 (6)	0.0013 (6)	0.0001 (6)
C5	0.0360 (7)	0.0366 (7)	0.0328 (7)	0.0086 (5)	−0.0018 (5)	0.0087 (5)

### Geometric parameters (Å, º)

**Table:**

O1—C5	1.2234 (16)	C2—C5	1.5504 (15)
N1—C5	1.3243 (17)	C3—H3A	0.9600
N1—H1A	0.92 (2)	C3—H3B	0.9600
N1—H1B	0.88 (2)	C3—H3C	0.9600
N2—C1	1.1395 (18)	C4—H4A	0.9600
C1—C2	1.4774 (17)	C4—H4B	0.9600
C2—C3	1.5356 (18)	C4—H4C	0.9600
C2—C4	1.5438 (18)		
			
C5—N1—H1A	114.6 (12)	C2—C3—H3C	109.5
C5—N1—H1B	124.9 (12)	H3A—C3—H3C	109.5
H1A—N1—H1B	120.5 (18)	H3B—C3—H3C	109.5
N2—C1—C2	178.86 (14)	C2—C4—H4A	109.5
C1—C2—C3	109.07 (10)	C2—C4—H4B	109.5
C1—C2—C4	109.37 (10)	H4A—C4—H4B	109.5
C3—C2—C4	110.22 (10)	C2—C4—H4C	109.5
C1—C2—C5	111.37 (9)	H4A—C4—H4C	109.5
C3—C2—C5	109.91 (10)	H4B—C4—H4C	109.5
C4—C2—C5	106.88 (10)	O1—C5—N1	123.35 (11)
C2—C3—H3A	109.5	O1—C5—C2	118.09 (10)
C2—C3—H3B	109.5	N1—C5—C2	118.50 (10)
H3A—C3—H3B	109.5		
			
N2—C1—C2—C3	78 (8)	C4—C2—C5—O1	76.58 (15)
N2—C1—C2—C4	−42 (8)	C1—C2—C5—N1	18.55 (16)
N2—C1—C2—C5	−160 (8)	C3—C2—C5—N1	139.54 (12)
C1—C2—C5—O1	−164.02 (12)	C4—C2—C5—N1	−100.85 (14)
C3—C2—C5—O1	−43.03 (15)		

### Hydrogen-bond geometry (Å, º)

**Table:**

*D*—H···*A*	*D*—H	H···*A*	*D*···*A*	*D*—H···*A*
N1—H1*A*···O1^i^	0.92 (2)	2.07 (2)	2.9714 (18)	168.2 (18)
N1—H1*B*···N2^ii^	0.874 (18)	2.328 (18)	3.166 (2)	160.8 (19)

Symmetry codes: (i) −*x*, −*y*+1, −*z*; (ii) −*x*+1, −*y*+1, −*z*+1.
